# AIDS and AAA in Egypt?

**DOI:** 10.3201/eid0204.960419

**Published:** 1996

**Authors:** R J Littman, D M Morens


7. Miller RL, Armelagos GJ, Ikram S, de Jonge N, Krijger FW, Deelder AM. Paleoepidemiology of *Schistosoma* infection in mummies. Br Med J 1992;304:555-6.
8. Capron A, Dessaint JP, Capron M, Ouma JH, Butterworth AE. Immunity to schistosomes: Progress toward a vaccine. Science 1987;238:1065-72.
9. Gryseels B, Stelma FF, Talla I, van Dam GJ, Polman K, Sow S, et al. Epidemiology, immunology and chemotherapy of *Schistosoma mansoni* infections in a recently exposed community in Senegal. Trop Geogr Med 1994;46:209-19.
10. El Alamy MA, Cline BL. Prevalence and intensity of *Schistosoma haematobium* and *S. mansoni* infection in Qalyub, Egypt. Am J Trop Med Hyg 1977;26:470-2.

### AIDS and *ĀĀĀ* in Egypt?

**To the Editor:** A recent letter concerning Egyptian hieroglyphs on the disease *ĀĀĀ* asks if this disease could be AIDS or an HIV-associated condition prevalent in Egypt during the time of the pharaohs (1). We believe this possibility is highly unlikely. Aside from conflicts with current thought on the origin and evolution of lentiviruses, there is a problem of linguistic interpretation. The initial hieroglyph in the series of hieroglyphs comprising the word *ĀĀĀ*, a picture of a discharging phallus, is a "determinative," indicating the class or category to which the word belongs. Although scholars once took this determinative to indicate a phallic connection with disease, even suggesting that *ĀĀĀ* meant hematuria, consistent with schistosomiasis (2,3), it was later proposed that the determinative meant semen or poison, reflecting the Egyptian concept that diseases may be transmitted by an evil spirit in the form of an incubus, impregnating a victim with poisonous semen. This interpretation is now generally accepted (4,5). The phallus-with-discharge thus came to indicate a deadly disease, and *ĀĀĀ* a poisonous disease-causing substance introduced into the body by magic. The word *ĀĀĀ* is used elsewhere in the Egyptian medical papyri in other contexts, such as "*ĀĀĀ* of the heart" and "*ĀĀĀ* of the belly and heart," and is not known to have been used in connection with the bladder or genitalia. While the determinative meaning may not be absolutely established, it is clear from its usage in other contexts that the phallus-with-discharge determinative can indicate fatal or serious illness. The notion that the phallus-with-discharge determinative refers to sexually transmitted disease is not consistent with its usage. To further argue that *ĀĀĀ* represents AIDS or HIV disease is not justified by the linguistic evidence. Without further archaeologic or inscriptional evidence, we would doubt that HIV circulated in ancient Egypt.

**Robert J. Littman and David M. Morens**
University of Hawaii, Honolulu, Hawaii

### References

1. Ablin RJ. Déjà vu in ancient Egypt? Emerging Infectious Diseases 1996;2:242.
2. Ebbell B. The Papyrus Ebers. London: Oxford University Press, 1937.
3. Jonckheere F. Une Maladie Égyptienne, l'Hématurie Parasitaire. Bruxelles: Fondation Égyptologique Reine Élizabeth, 1944.
4. von Deines H, Westendorf W. Wörterbuch der medizinischen Texte. Erste Hälfte. Berlin: Akademie-Verlag, 1961;7(Part 1):129.
5. Nunn JF. Chapter 3. Concepts of anatomy, physiology and pathology. Chapter 4. The pattern of disease. Chapter 5. Magic and religion in medicine. In: Nunn JF: Ancient Egyptian Medicine. London: British Museum Press, 1966:42-63,64-95,96-112.

